# Effectiveness of hydrogen peroxide fumigation using an automated fogging system for environmental management in educational institutions

**DOI:** 10.1038/s41598-026-55618-2

**Published:** 2026-05-28

**Authors:** Ewelina Kruszewska, Piotr Czupryna, Diana Martonik, Anna Moniuszko-Malinowska

**Affiliations:** 1 Department of Infectious Diseases and Neuroinfections, Medical University of Bialystok, Zurawia 14, Bialystok, 15-540 Poland; 2 Department of Infectious Diseases and Hepatology, Medical University of Bialystok, Zurawia 14, Bialystok, 15-540 Poland

**Keywords:** Environmental sciences, Health care, Microbiology

## Abstract

The presence of microbial contamination on surfaces in early childhood education facilities poses a significant public health risk. Traditional cleaning methods often fail to fully eliminate pathogens, necessitating the development of more effective disinfection strategies. Hydrogen peroxide fumigation has emerged as a promising approach due to its broad-spectrum antimicrobial activity, environmental safety, and ability to penetrate biofilms, with potential implications for broader environmental management strategies. This study aimed to evaluate the effectiveness of a novel, fully automated fogging cabinet that generates hydrogen peroxide dry mist for disinfecting toys in educational settings. The most frequently detected bacteria before disinfection included *Micrococcus luteus* and *Staphylococcus hominis*, accounting for 30.3–40% and 22.8–32.2% of total bacterial contamination, respectively. The overall disinfection effectiveness was 97.8%, 97%, and 98.7% across the three tested facilities (*p* < 0.001). Near-complete eradication (≥ 99%) was observed for *Acinetobacter lwoffii*, *Pseudomonas luteola*, and *Staphylococcus haemolyticus*. The findings corroborate previous research on the efficacy of hydrogen peroxide disinfection, including studies on public transportation and healthcare settings. The project, conducted with MEDILAB Sp. z o.o., resulted in the development of a portable, automated fogging cabinet for disinfecting toys. A pilot study was carried out in three educational institutions in Białystok, Poland, where 180 microbiological samples were collected from plastic toys before and after disinfection. The process used a hydrogen peroxide-based mist generated inside the cabinet over 30 min. Samples were incubated, and bacterial colonies were identified using MALDI-TOF mass spectrometry (VITEK MS). Statistical analysis confirmed the effectiveness of the disinfection method. This study demonstrates that the newly developed hydrogen peroxide-based fogging cabinet is an effective and reliable method for mitigating bacterial contamination on toys in early childhood education facilities. Its automated, contact-free application minimizes human error and ensures consistent disinfection, supporting the broader implementation of hydrogen peroxide fumigation as a standardized disinfection strategy in educational and other high-contact environments. Furthermore, the proposed automated fogging system represents an innovative approach by eliminating human error and ensuring uniform disinfectant distribution.

## Introduction

Amid rising demands for improved sanitary safety, conventional disinfection methods often prove inadequate^1–3^. Surface contamination in educational institutions poses significant health risks^4–7^, necessitating effective solutions with both local and global applicability. This study evaluates the efficacy of a novel automated fogging system that generates a hydrogen peroxide dry mist, capable of uniformly disinfecting hard-to-reach areas. The technology’s potential for broader environmental management is discussed in the context of evolving public health challenges.

Objects commonly encountered in public environments can serve as reservoirs for diverse range of pathogens, including bacteria and viruses. These microorganisms have the capacity to persist on surfaces for prolonged time and disseminate through contact with surfaces or human hands^4,8,9^.

Despite existing preventive measures, contact-based cross-transmission remains a significant source of infection for individuals who during the day spend substantial time indoors^1^. Students in educational settings are particularly susceptible to upper respiratory and gastrointestinal infections^5,6^. This increased vulnerability stems from children’s developing immune systems and behavior, which provides multiple ways to be exposed to germs and infections. Young children frequently fail to adhere to proper hygiene practices, such as thorough hand washing or covering their mouths when coughing^7^. These behaviors result in increased illness rates among children and staff in educational institutions, leading to reduced productivity for parents and educators, who must take leave from work. Additionally, infections increase the risk of hospitalization, resulting in higher healthcare expenses. To address this issue, educational institutions are implementing preventive strategies including regular manual cleaning of frequently touched surfaces.

Traditional cleaning methods are insufficient to completely eliminate pathogen contamination on surfaces and objects^1,8,10^. Current disinfection challenges include biofilms that resist routine cleaning procedures, and the need for biocides that are both efficacious and safe for users and materials^8^. Hydrogen peroxide, initially synthesized in 1818 by Louis Jacques Thénard, is emerging as a promising solution owing to its broad-spectrum sterilization activity and compatibility with surface materials^11^. Its decomposition products are oxygen and water, rendering hydrogen peroxide a safe disinfectant for human health and the environment^12^. Hydrogen peroxide disinfection gained prominence in the 1980 s and continues to be widely used in contemporary practice. The substance can be applied directly as a disinfectant or sterilizer in aqueous solutions with concentrations ranging from 3 to 9%, or it can be combined with other disinfecting agents such as peracetic acid in water, or utilized as a gas in vapor or aerosol form^13,14^. Hydrogen peroxide in the form of dry mist, delivered by specialized equipment, can supplement fundamental cleaning procedures. Fumigation with this substance is an increasingly prevalent disinfection method employed in hospitals for room sterilization and medical equipment disinfection, as well as in public transportation and agriculture, among other applications^2,15–18^.

Hydrogen peroxide fumigation is a sophisticated and effective disinfection method that has become an essential component of public health strategies aimed at combating pathogens. The complementary nature of its antimicrobial properties, coupled with its environmental safety, suggests an increasing role for public health management. However, as with any disinfection strategy, a consideration of its advantages and limitations, in parallel with comparisons with alternative methods, is essential for making informed decisions regarding the implementation of hygiene protocols. Hydrogen peroxide fumigation offers several advantages that make it a practical disinfection option in public buildings. One of the main advantages of using hydrogen peroxide is its ability to penetrate porous materials and access difficult-to-reach areas, improving overall efficacy in killing pathogens. Traditional surface disinfectants often fail to achieve similarly complete infiltration, particularly in complex environments where pathogens may reside in microenvironments that prevent direct contact. This makes hydrogen peroxide particularly valuable in settings such as hospitals, schools and public transport systems where thorough disinfection is fundamental^3,10,14,19,20^.

The persistent presence of pathogens on surfaces in public environments poses a significant health risk, particularly in educational settings where children are vulnerable to infections. While traditional cleaning methods may not suffice, hydrogen peroxide, particularly in its dry mist form, offers a promising solution for effective disinfection^21^. Its broad-spectrum efficacy, safety profile, and ability to penetrate biofilms make it an ideal candidate for implementation in schools to reduce the transmission of infectious agents. As educational institutions continue to seek effective strategies to enhance hygiene and protect the health of students and staff, the adoption of hydrogen peroxide dry mist technology could play a pivotal role in mitigating the risks associated with surface contamination.

**Fig. 1:**
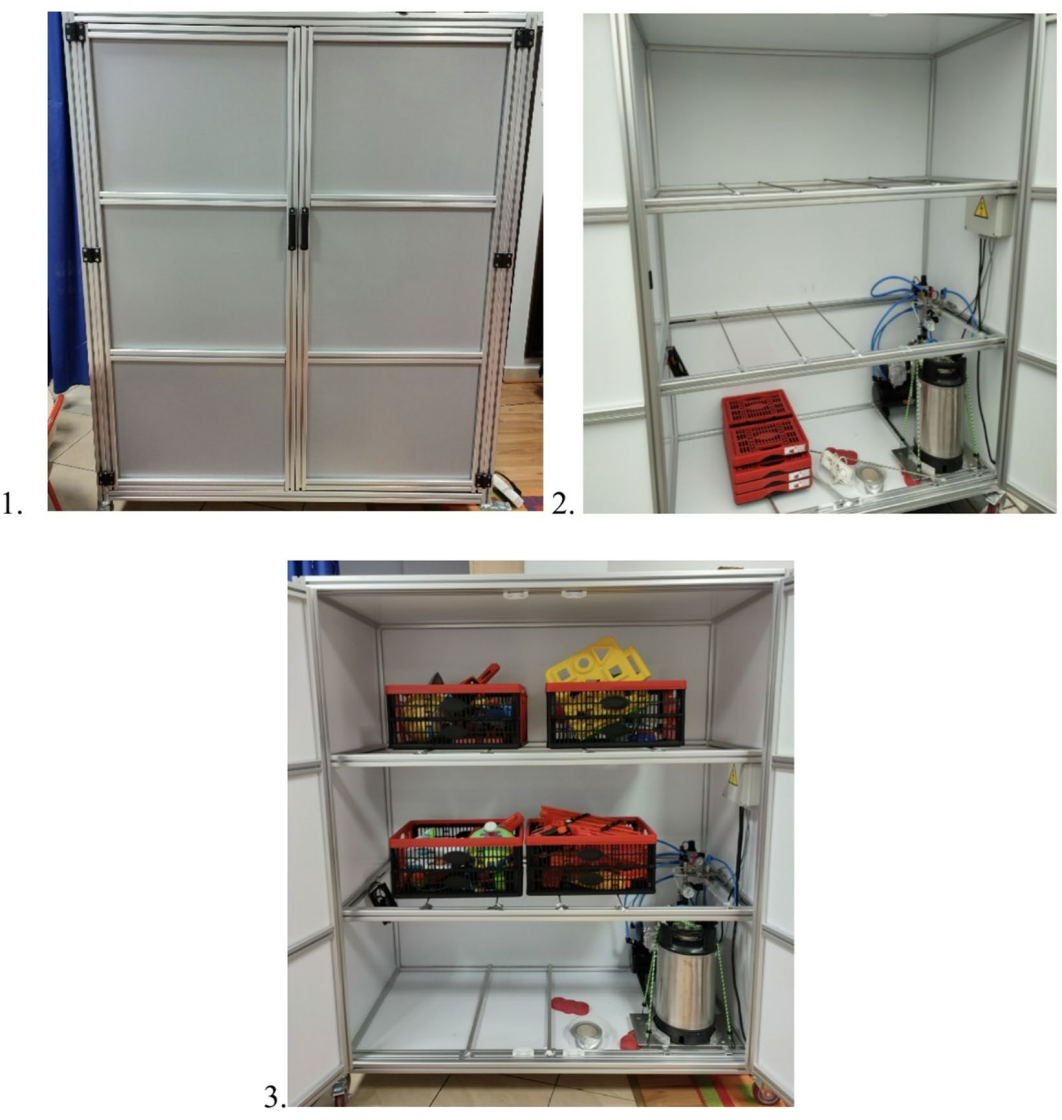
1, 2, 3 The fogging cabinet used in the study.

The aim of the study was to evaluate the effectiveness of a new fogging cabinet as presented on Fig. 1, that generates hydrogen peroxide in the form of a dry mist as a disinfection method against bacterial contamination on toys in educational centers.

## Methods

This study was designed as an implementation-oriented pilot investigation to evaluate the effectiveness of hydrogen peroxide dry mist disinfection in real-world educational settings. A pilot study was conducted in one nursery (Educational institution A) and two kindergartens in Bialystok, Poland (Educational institution B and C), where 30 toys were disinfected in each facility. Microbiological samples were collected from plastic toys with smooth surfaces, 180 impressions in total.

The system used in the present study was developed as an automated, closed-cabinet solution, minimizing operator exposure and reducing the risk of human error, which is consistent with current trends in “no-touch” disinfection technologies^2^. The study protocol, including the use of the device, was approved by the Bioethics Committee of the Medical University of Bialystok (approval no. R-I-002/344/2018). In addition, permission to conduct the study was obtained from the administrations of all participating educational institutions.

Prior to the initiation of the study, the staff of the participating educational institutions were formally informed about the objectives, procedures, and schedule of the project. The study was conducted outside regular educational hours, specifically after daily activities in nurseries and kindergartens, in order to ensure that neither children nor staff were present during the disinfection process.

Each institution had a designated room with a fumigation cabinet, where toys were brought. The fumigation procedure was conducted by the researcher using a hydrogen peroxide-based dry mist system in a dedicated disinfection cabinet.

Following the disinfection process, the rooms and toys were thoroughly ventilated for several hours to ensure complete dissipation of residual disinfectant. All procedures were consistently performed on Fridays, allowing extended ventilation over the weekend. Consequently, children were re-exposed to the disinfected toys no earlier than three days after the procedure, thereby ensuring a high level of safety and eliminating the risk of direct exposure to the disinfectant.

The fogging system used in this study was developed as a research prototype in collaboration with MEDILAB Sp. z o.o. and consisted of a custom-designed, enclosed cabinet equipped with an aerosol-generating device intended for the application of hydrogen peroxide-based disinfectants. The aerosol generator, supplied by MEDILAB Sp. z o.o., operated on the principle of ultra-low volume (ULV) fogging, producing a fine dry mist composed of microdroplets, enabling uniform distribution of the disinfectant within the closed chamber. The design and configuration of the fogging cabinet are presented in Fig. 1 (1–3).

A ready-to-use hydrogen peroxide-based formulation (O2 SAFE 7.4) was used as the disinfecting agent. The design and operational principles of the system were based on established hydrogen peroxide fumigation technologies described in the literature, which have demonstrated high antimicrobial efficacy and safety in enclosed environments^2,15,21^.

The application of dry mist hydrogen peroxide systems has been shown to ensure uniform distribution of the disinfectant, including penetration into hard-to-reach areas, while maintaining a favorable safety profile due to its decomposition into water and oxygen^14,22^. Similar fogging and fumigation approaches have been successfully implemented in educational facilities and other public environments, confirming their suitability for infection control purposes^14,20^.

Before disinfection, microbiological samples were collected using Tryptic Soy Agar (TSA) contact plates with a surface area of 25 cm² (manufacturer: Poland). The plates were applied to the toy surfaces using a BIOMAXIMA applicator, a device designed to ensure standardized and reproducible sampling conditions by maintaining a constant contact force corresponding to approximately 500 g (≈ 4.9 N) and a fixed contact time of 10 s. Sampling sites were not selected randomly but were deliberately chosen on flat, easily accessible surfaces of the toys to ensure full and uniform contact between the agar plate and the sampled area. This approach allowed for standardized and reproducible sampling conditions. Irregular or hard-to-reach parts were excluded due to technical limitations of the contact plate method. Each sample corresponded to a defined surface area (25 cm²), and results were expressed as CFU/25 cm². All samples were collected under aseptic conditions.

The fumigation process was carried out using the newly developed, fully automated fogging generator designed for disinfecting toys in educational centers. This cabinet generates a mist consisting of microscopic droplets (5–10 μm). A ready-to-use O2 SAFE 7.4 solution based on hydrogen peroxide was used for disinfection, with the aerosol generator placed inside the fogging cabinet. The misting process lasted 30 min. This exposure time was selected based on device operational parameters and published data on hydrogen peroxide fumigation systems, where effective microbial reduction is typically achieved within exposure times ranging from approximately 20 to 60 min, depending on system configuration and environmental conditions^2,19,23^.

Half an hour after the disinfection process samples were collected from the same predefined surface areas on each toy that had been sampled prior to disinfection, ensuring a paired sampling design and allowing for direct comparison of microbial load before and after the intervention. The agar plates were incubated for 48 h at a temperature of 35℃ ± 2℃, after which the number of bacterial colonies per plate was counted and expressed in CFU/25 cm².

Bacterial colony isolation was performed on Columbia agar plates with 5% sheep blood, followed by aerobic incubation for 24 h at 35℃ ± 2℃, to ensure optimal recovery and differentiation of a broad spectrum of clinically relevant bacteria, including fastidious organisms. This enriched, non-selective medium supports the growth of Gram-positive and Gram-negative bacteria and enables assessment of hemolytic activity, which facilitates preliminary differentiation prior to identification.

Following incubation, microorganism identification was conducted using the VITEK MS mass spectrometry system (bioMérieux) based on matrix-assisted laser desorption/ionization time-of-flight (MALDI-TOF) technology^24–26^. For analysis, a single, well-isolated bacterial colony was aseptically transferred onto a disposable target plate using a sterile applicator. No chemical disinfection of the colony was performed prior to analysis, as standard MALDI-TOF protocols rely on direct transfer under sterile conditions to avoid contamination while preserving protein integrity^24,25^.

Subsequently, 1 µL of matrix solution containing α-cyano-4-hydroxycinnamic acid (CHCA) was applied to the sample spot. The CHCA matrix consisted of a saturated solution of α-cyano-4-hydroxycinnamic acid dissolved in a mixture of organic solvent (acetonitrile) and water with the addition of trifluoroacetic acid (TFA), which facilitates protein extraction and co-crystallization^24,26^. After air drying at room temperature, the target plate was inserted into the VITEK MS instrument. Microbial identification was achieved by comparing the obtained mass spectra with reference spectra stored in the VITEK MS database^24–26^.

Data were subjected to statistical analysis using Statistica 13.0 software (StatSoft Inc., Krakow, Poland), with p-values < 0.05 considered statistically significant. Wilcoxon signed-rank test was used to assess the disinfection effectiveness.

## Results

The most frequently occurring bacteria across all three educational facilities were *Micrococcus luteus* (*M. luteus*) and *Staphylococcus hominis* (*S. hominis*). The prevalence of *M. luteus* was 40%, 39.2%, and 30.3% in educational facilities A, B, and C, respectively. *S. hominis* accounted for approximately one-third of the bacteria present in educational facilities A and C (32.2% and 31.5%, respectively), while in facility B, it constituted 22.8%.

*Staphylococcus epidermidis* (*S. epidermidis*) was most prevalent in educational facility B (16.7%), whereas its occurrence in facilities A and C did not exceed 5%. *Bacillus flexus* (*B. flexus*) accounted for 10.4% of the bacteria found in facility C, while in facility B, its presence was only 0.6%. Meanwhile, *Staphylococcus warneri* (*S. warneri*) made up 6.9% of the bacteria in facility C but only 0.7% in facility A.

**Fig. 2:**
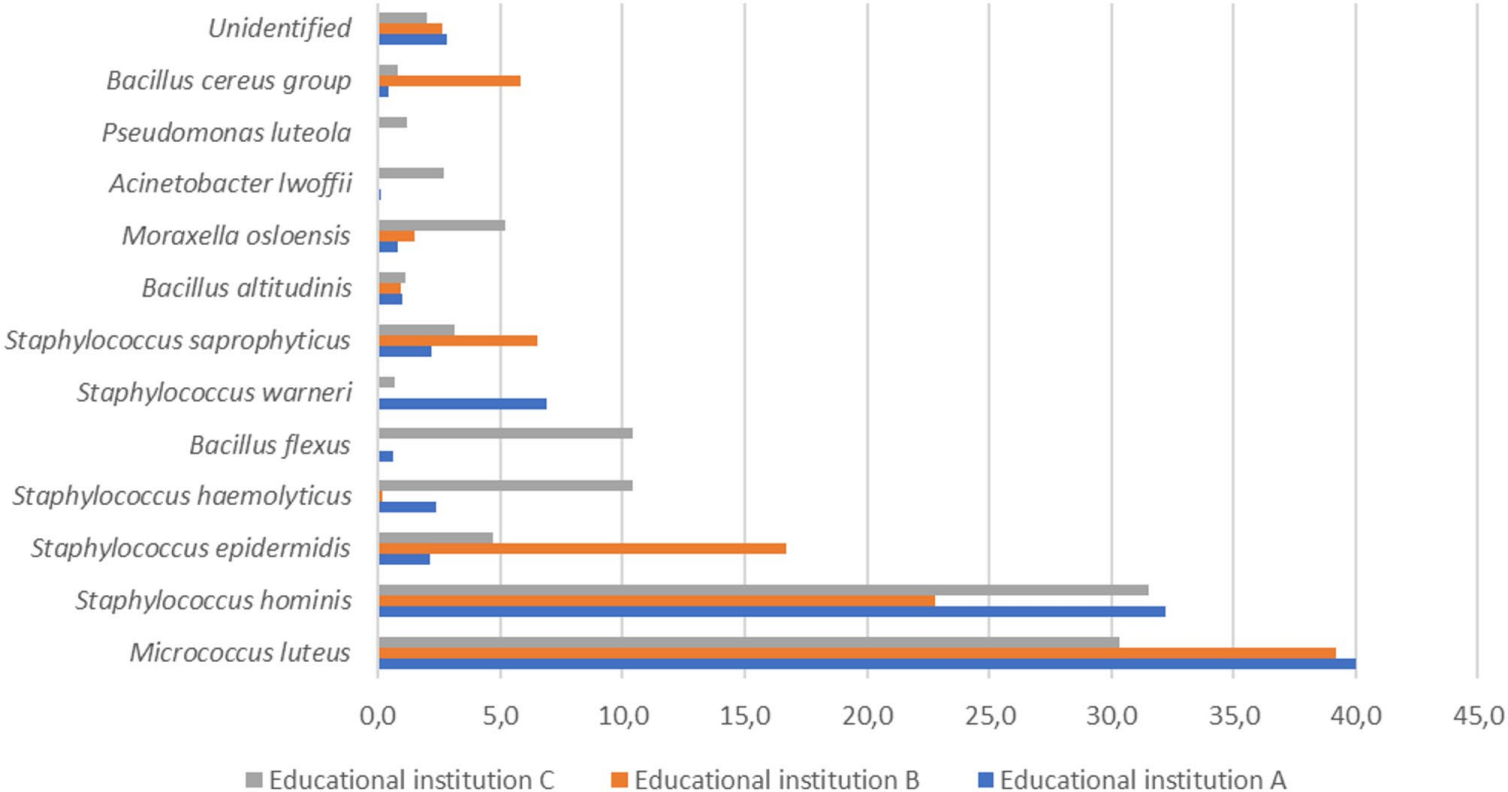
Frequency of occurrence of bacteria on toys in individual educational institutions.

Detailed data on bacterial prevalence are presented in Table 1; Fig. 2.

**Table 1:** Frequency of occurrence of bacteria on toys in individual educational institutions.

	Frequency of occurrence [%]		
	Educational institution A	Educational institution B	Educational institution C
*Micrococcus luteus*	40	39.2	30.3
*Staphylococcus hominis*	32.2	22.8	31.5
*Staphylococcus epidermidis*	2.1	16.7	4.7
*Staphylococcus haemolyticus*	2.4	0.2	10.4
*Bacillus flexus*	0.6	-	10.4
*Staphylococcus warneri*	6.9	-	0.7
*Staphylococcus saprophyticus*	2.2	6.5	3.1
*Bacillus altitudinis*	1	0.9	1.1
*Moraxella osloensis*	0.8	1.5	5.2
*Acinetobacter lwoffii*	0.1	-	2.7
*Pseudomonas luteola*	-	-	1.2
*Bacillus cereus* group	0.4	5.8	0.8
Unidentified	2.8	2.6	2.0

The study demonstrated significant efficacy in microbial inactivation using the newly developed toy disinfection cabinet. The overall disinfection effectiveness was 97.8% in educational facility A (*p* < 0.001), 97% in facility B (*p* < 0.001), and 98.7% in facility C (*p* < 0.001).

The highest bacterial reduction (100%) was observed for *Acinetobacter lwoffii* (*A. lwoffii*), *Pseudomonas luteola* (*P. luteola*), and *Staphylococcus haemolyticus* (*S. haemolyticus*) in facility C, as well as for *Bacillus altitudinis* (*B. altitudinis*) in facility A. The highest reduction of *Bacillus cereus* (*B. cereus*) was noted in facility B, reaching 98.6%.

Detailed data on the effectiveness of disinfection for specific bacterial strains, according to location, are presented in Table 2.

**Table 2:** Disinfection effectiveness of individual bacterial strains on toys, taking into account location.

	Educational institution A		Educational institution B		Educational institution C	
	Disinfection effectiveness [%]	*p*	Disinfection effectiveness [%]	*p*	Disinfection effectiveness [%]	*p*
Total value	97.8	< 0.001	97	< 0.001	98.7	< 0.001
*Staphylococcus hominis*	99.3	< 0.001	96.5	< 0.001	99.8	< 0.001
*Staphylococcus epidermidis*	91.9	0.045	94.8	< 0.001	96.7	0.001
*Micrococcus luteus*	96.2	< 0.001	97.8	< 0.001	98.2	< 0.001
*Staphylococcus warneri*	98.3	0.017	-	-	-	-
*Bacillus altitudinis*	100	0.002	-	-	-	-
*Bacillus cereus* group	-	-	98.6	< 0.001	-	-
*Moraxella osloensis*	-	-	-	-	97	0.017
*Acinetobacter lwoffii*	-	-	-	-	100	0.011
*Staphylococcus saprophyticus*	-	-	-	-	97.5	0.007
*Pseudomonas luteola*	-	-	-	-	100	0.011
*Staphylococcus haemolyticus*	-	-	-	-	100	0.024
Unidentified	97.9	0.002	93.9	0.004	-	-

## Discussion

The findings of this study demonstrate the high efficacy of hydrogen peroxide fumigation in reducing bacterial contamination on toys in early childhood education facilities. The newly developed fogging cabinet achieved overall disinfection effectiveness rates of 97.8%, 97%, and 98.7% in the three tested educational institutions, confirming the potential of dry mist hydrogen peroxide as a robust and reliable disinfection method. Although the observed disinfection efficacy (~ 97%) indicates a substantial reduction in microbial load, it should not be interpreted as complete elimination of all microorganisms. In environments contaminated with hazardous or highly resistant microbial isolates, even a small residual population may pose a potential risk of persistence and subsequent transmission. Previous studies have demonstrated that hydrogen peroxide-based fumigation systems typically achieve high-level disinfection rather than sterilization, with effectiveness depending on factors such as organism type, biofilm presence, and environmental conditions^2,27^.

The level of reduction observed in the present study is consistent with previously reported efficacy ranges for hydrogen peroxide fumigation systems, which typically achieve microbial reductions exceeding 90–99% under varying environmental conditions^2,19,27^. In comparison to studies conducted in controlled laboratory or healthcare environments, the results obtained here reflect real-world conditions, where variability in contamination levels and surface characteristics may influence overall effectiveness. This indicates that the evaluated dry mist system maintains high performance even outside controlled settings.

From a practical perspective, a ~ 97% reduction may be considered acceptable for non-critical environments such as educational settings, where the primary goal is to reduce microbial burden and transmission risk rather than achieve sterility. However, in high-risk settings (e.g., healthcare or laboratory environments), higher log-reduction thresholds (≥ 99.9% or complete sterilization) are generally required. Therefore, the achieved level of disinfection should be interpreted in the context of the intended application, and where necessary, supplemented by repeated disinfection cycles or combined hygiene strategies. Similar considerations have been highlighted in previous studies, where acceptable disinfection thresholds were shown to depend strongly on the intended use of the environment and the associated level of infection risk^2,19^.

This limitation should be acknowledged when interpreting the results, as the persistence of a small fraction of microorganisms, particularly biofilm-forming or resistant strains, cannot be entirely excluded. These results underscore the critical importance of effective disinfection strategies in environments where bacterial transmission is a significant concern, particularly in educational settings.

Prior to disinfection, the study identified *Micrococcus luteus* and *Staphylococcus hominis* as the most prevalent bacterial species on toys, a microbial profile that is consistent with previous research on the pathogens commonly found on frequently touched surfaces in childcare facilities^4^. The high prevalence of these bacteria emphasizes the need for comprehensive and effective disinfection protocols to mitigate microbial transmission in educational environments.

Comparable microbial profiles have also been reported in studies conducted in similar environments, where skin-associated and environmental bacteria dominate surface contamination, further supporting the external validity of the present findings^4^.

The applied disinfection time (30 min) is consistent with previously reported hydrogen peroxide fumigation protocols. Studies on airborne and vaporized hydrogen peroxide systems indicate that effective microbial inactivation typically requires extended exposure times, usually ranging from 20 to 60 min, depending on system configuration, environmental conditions, and target microorganisms^2,19,23^. Shorter exposure times may result in incomplete microbial reduction, particularly in the case of more resistant organisms or in complex environments where uniform distribution of the disinfectant is required. Therefore, the selected exposure time represents a practical and literature-supported compromise between efficacy and operational feasibility.

In comparison with previously published data, the selected exposure time falls within the optimal range; however, some studies have reported increased efficacy with longer exposure durations, suggesting that further optimization of process parameters may yield even higher levels of microbial reduction in more demanding conditions.

In terms of bacterial reduction, our study demonstrated near-total eradication of several bacterial species, including *Acinetobacter lwoffii*, *Pseudomonas luteola*, and *Staphylococcus haemolyticus*. The observed efficacy aligns with prior studies indicating that hydrogen peroxide vapor effectively inactivates both Gram-positive and Gram-negative bacteria^27^. However, some variation in bacterial susceptibility was noted, with slightly lower reduction rates for *Staphylococcus epidermidis*, which can be attributed to the biofilm-forming properties of this bacterium, as reported by Farjami et al.^28^. The biofilm formation capability of *S. epidermidis* likely contributes to its resistance to disinfection efforts, highlighting the challenge of eradicating biofilm-associated microbes. Similar variability in susceptibility has been widely described in the literature, particularly in relation to biofilm-forming microorganisms, which are known to exhibit increased tolerance to disinfectants^28^. Differences between studies may also result from variations in initial contamination levels, environmental complexity, and microbial composition.

An important aspect of this study is the demonstration that hydrogen peroxide fumigation can effectively penetrate and disrupt biofilms, which are known to protect bacteria from standard disinfection methods. Previous studies have highlighted the ability of hydrogen peroxide to interact with bacterial biofilm components, thereby enhancing its bactericidal activity^28^. Our findings further support this, as we achieved over 90% reduction in *S. epidermidis*, a bacterium commonly associated with biofilm formation. This underscores the advantage of hydrogen peroxide fumigation over traditional surface disinfectants, particularly in overcoming biofilm-associated resistance.

When compared to manual cleaning and conventional disinfection methods, our study suggests that automated hydrogen peroxide fumigation offers a more consistent and thorough decontamination strategy. In a previous study^20^, the efficacy of peracetic acid combined with hydrogen peroxide was demonstrated in public transportation settings. While both methods were effective, the dry mist system developed in this study provides a fully automated, contact-free approach, reducing human error and ensuring even distribution of the disinfectant. Similarly, Bukłaha et al.^29^ evaluated the efficacy of various disinfectants in vehicle air conditioning systems, finding that peracetic acid stabilized with hydrogen peroxide effectively reduced microbial contamination in enclosed spaces. These studies support the broader application of hydrogen peroxide-based disinfection methods in public environments, highlighting their potential for use in settings beyond educational facilities.

In contrast to conventional manual cleaning, which is often associated with variability in application and incomplete surface coverage, automated fumigation systems provide more standardized and reproducible conditions, as also reported in previous studies^1,2^. However, it should be noted that the effectiveness of such systems remains dependent on prior removal of organic matter, and real-world conditions may limit performance compared to controlled environments.

Safety and environmental considerations are also essential when evaluating the suitability of hydrogen peroxide fumigation in childcare settings. Our study aligns with previous findings that low concentrations of hydrogen peroxide do not pose significant health risks to children or staff when used correctly^22^. The decomposition of hydrogen peroxide into water and oxygen further supports its suitability for educational environments, where non-toxic and residue-free disinfection is critical.

Despite its high efficacy, hydrogen peroxide fumigation systems have several limitations that should be considered. The effectiveness of the process depends on controlled environmental conditions, including proper enclosure and uniform distribution of the disinfectant^2,19^. In addition, material compatibility must be taken into account, as repeated exposure to hydrogen peroxide may affect certain sensitive surfaces^12,21^. Another important aspect is the need for adequate post-treatment ventilation to ensure complete removal of residual hydrogen peroxide and maintain user safety^22^. These factors should be carefully considered when implementing this disinfection method in practical settings.

Similar limitations have been reported in previous studies, in which environmental factors and operational parameters were identified as key determinants of disinfection effectiveness^2,19^.

The broad applicability of hydrogen peroxide fumigation is further supported by studies such as Sanguinet and Edmiston^30^, who demonstrated its role in reducing microbial bioburden in healthcare facilities, and Amodio et al.^21^, who highlighted its adjunctive role in disinfecting non-critical medical equipment. These findings suggest that hydrogen peroxide fumigation could be effectively implemented in various institutional settings, ranging from educational to healthcare environments.

Beyond educational facilities, this fogging system holds promise for deployment in other sectors requiring rigorous hygiene standards, such as public transportation, industrial settings, and healthcare institutions. In light of global environmental challenges and the increasing need for effective infection control, the implementation of automated disinfection technologies, such as hydrogen peroxide fumigation, could establish new standards for infection prevention across a wide range of settings.

One primary limitation of this study is its localized scope. Future research should include multi-regional studies to validate the generalizability of these findings and explore the potential for broader implementation across various sectors.

## Conclusions

In conclusion, the pilot study demonstrates that the newly developed automated fogging cabinet for disinfecting toys using hydrogen peroxide dry mist is an effective solution for reducing microbial contamination in educational settings. The high disinfection efficacy, combined with the safety and environmental benefits of hydrogen peroxide, positions this technology as a valuable tool in the ongoing effort to protect children’s health in communal spaces. As educational institutions continue to prioritize hygiene and infection control, the integration of such innovative disinfection methods will be crucial in mitigating the risks associated with pathogen transmission.

## Data Availability

The datasets generated and analyzed during the current study are publicly available in the Zenodo repository at: https://doi.org/10.5281/zenodo.19690706.

## Abbreviations

*A. lwoffii*: *Acinetobacter lwoffii*
*B. altitudinis*: *Bacillus altitudinis*
*B. cereus*: *Bacillus cereus*
*B. flexus*: *Bacillus flexus*
CFU: Colony Forming Units
CHCA: α-Cyano-4-hydroxycinnamic acid
MALDI-TOF: Matrix-Assisted Laser Desorption/Ionization Time-of-Flight
*M. luteus*: *Micrococcus luteus*
MS: Mass Spectrometry
*P. luteola*: *Pseudomonas luteola*
*S. epidermidis*: *Staphylococcus epidermidis*
*S. haemolyticus*: *Staphylococcus haemolyticus*
*S. hominis*: *Staphylococcus hominis*
*S. saprophyticus*: *Staphylococcus saprophyticus*
*S. warneri*: *Staphylococcus warneri*
TFA: Trifluoroacetic acid
TSA: Tryptic Soy Agar
VITEK MS: VITEK mass spectrometry system (bioMérieux)
µm: Micrometr
O2 SAFE 7.4: Hydrogen peroxide-based disinfectant solution
